# Household Coverage with Adequately Iodized Salt Varies Greatly between Countries and by Residence Type and Socioeconomic Status within Countries: Results from 10 National Coverage Surveys^1^,^2^,^3^

**DOI:** 10.3945/jn.116.242586

**Published:** 2017-04-12

**Authors:** Jacky M Knowles, Greg S Garrett, Jonathan Gorstein, Roland Kupka, Ruth Situma, Kapil Yadav, Rizwan Yusufali, Chandrakant Pandav, Grant J Aaron, the Universal Salt Iodization Coverage Survey Team

**Affiliations:** 4 Global Alliance for Improved Nutrition, Geneva, Switzerland;; 5 Sajilo Solutions International, Seattle, WA;; 6 Nutrition Section, UNICEF, New York, NY; and; 7 Centre for Community Medicine, All India Institute of Medical Sciences, New Delhi

**Keywords:** salt iodization, coverage survey, USI, iodine, iodine deficiency, micronutrient, elimination of IDD

## Abstract

**Background:** Household coverage with iodized salt was assessed in 10 countries that implemented Universal Salt Iodization (USI).

**Objective:** The objective of this paper was to summarize household coverage data for iodized salt, including the relation between coverage and residence type and socioeconomic status (SES).

**Methods:** A review was conducted of results from cross-sectional multistage household cluster surveys with the use of stratified probability proportional to size design in Bangladesh, Ethiopia, Ghana, India, Indonesia, Niger, the Philippines, Senegal, Tanzania, and Uganda. Salt iodine content was assessed with quantitative methods in all cases. The primary indicator of coverage was percentage of households that used adequately iodized salt, with an additional indicator for salt with some added iodine. Indicators of risk were SES and residence type. We used 95% CIs to determine significant differences in coverage.

**Results:** National household coverage of adequately iodized salt varied from 6.2% in Niger to 97.0% in Uganda. For salt with some added iodine, coverage varied from 52.4% in the Philippines to 99.5% in Uganda. Coverage with adequately iodized salt was significantly higher in urban than in rural households in Bangladesh (68.9% compared with 44.3%, respectively), India (86.4% compared with 69.8%, respectively), Indonesia (59.3% compared with 51.4%, respectively), the Philippines (31.5% compared with 20.2%, respectively), Senegal (53.3% compared with 19.0%, respectively), and Tanzania (89.2% compared with 57.6%, respectively). In 7 of 8 countries with data, household coverage of adequately iodized salt was significantly higher in high- than in low-SES households in Bangladesh (58.8% compared with 39.7%, respectively), Ghana (36.2% compared with 21.5%, respectively), India (80.6% compared with 70.5%, respectively), Indonesia (59.9% compared with 45.6%, respectively), the Philippines (39.4% compared with 17.3%, respectively), Senegal (50.7% compared with 27.6%, respectively) and Tanzania (80.9% compared with 51.3%, respectively).

**Conclusions:** Uganda has achieved USI. In other countries, access to iodized salt is inequitable. Quality control and regulatory enforcement of salt iodization remain challenging. Notable progress toward USI has been made in Ethiopia and India. Assessing progress toward USI only through household salt does not account for potentially iodized salt consumed through processed foods.

## Introduction

Iodine deficiency is one of most important causes of preventable mental impairment around the world; inadequate thyroid hormone production of iodine also causes many other adverse effects on growth and development (1). Many of these adverse outcomes, collectively referred to as iodine deficiency disorders, result from the effects of iodine deficiency on fetal brain development during early pregnancy (2). Iodine deficiency can be effectively and inexpensively prevented by iodizing all salt for human and animal consumption [known as Universal Salt Iodization (USI)^9^] (3, 4). Since the early 1990s, a global effort, supported by international agencies and donors—most notably UNICEF—in partnership with national governments, salt industries, and academia, has resulted in a large increase in the percentage of the world’s population consuming adequately iodized salt (considered to be salt with ≥15 mg I/kg), from <20% in 1990 (5) (number of countries with data not mentioned) to 75% in 2014 (6) (98 countries with data from 2000 to 2013). In line with this increase, the number of countries with iodine deficiency (defined as a national median urinary iodine concentration of <100 μg/L in school-age children or, where data for children are unavailable, in women of reproductive age) decreased from >110 (of 121 countries with data) to 25 (of 155 countries with data) between 1993 and 2015 (7).

In 2008, with a grant from the Bill & Melinda Gates Foundation, the Global Alliance for Improved Nutrition and UNICEF formed the USI Partnership Project to intensify business-oriented efforts toward the global elimination of iodine deficiency. The goal of the Partnership Project was to increase household iodized salt coverage in 13 priority countries in order to achieve a combined household coverage of 85%. The 13 initial project countries were Bangladesh, China (7 provinces), Egypt, Ethiopia, Ghana, India, Indonesia, Niger, Pakistan, the Philippines, Russia, Senegal, and Ukraine. The total population of these countries at the start of the project was 2.3 billion. Support to efforts in Russia and Ukraine finished in 2012, earlier than in the other 11 countries. Countries were selected based on the size of the population in households without access to adequately iodized salt or on the potential for supporting effective salt industry change, in particular where a large proportion of salt was produced at small-scale production sites. At the start of the Partnership Project, national household coverage of adequately iodized salt was used as the key performance indicator to assess national progress toward USI and as a proxy for achievement of optimal population iodine nutrition.

National point estimates of household salt coverage based on field test methods were helpful to track progress after the initial implementation of salt iodization in the early 1990s. However, it has become increasingly clear that these data do not provide sufficient information to assess the quality of salt iodization or why iodization may have reached a plateau at levels well below 70% in some countries and subnational areas. This type of information is required to target national programs aiming to achieve optimal iodine nutrition for the whole population and to refine subnational approaches as needed (8). For example, intracountry disparities in household coverage of adequately iodized salt based on socioeconomic status (SES) and residence type have been shown to be pronounced (9).

The main objective of this paper is to summarize updated household iodized and adequately iodized salt coverage data from surveys conducted in 8 of the Partnership Project countries (Bangladesh, Ethiopia, Ghana, India, Indonesia, Niger, the Philippines, and Senegal) during the 2013–2015 period, together with data obtained from 2 national Fortification Assessment Coverage Toolkit surveys (10) in Tanzania and Uganda in 2015. In addition to presenting national data, the paper assesses the relation between household iodized and adequately iodized salt coverage and SES and urban or rural residence type.

Information presented in this paper was sourced from national survey reports [Bangladesh, India, and Senegal (11–13)] and from other documentation of survey outcomes or personal communication with the survey principal investigator (Ethiopia, Ghana, Indonesia, Niger, the Philippines, Tanzania and Uganda).

## Methods

### 

#### Survey design.

The surveys were cross-sectional multistage cluster surveys, all of which used a stratified design, with selection of primary sampling units within each stratum or domain based on probability proportional to size methodology, followed by systematic random sampling of a segment (where needed) and then of the required number of households within each primary sampling unit.

All surveys were designed to be representative of the population of the areas in which they took place. The iodine surveys conducted with Partnership Project technical support in Bangladesh, Ghana, and Senegal were designed to provide representative information by programmatically relevant domains, to provide an evidence base for the design of future strategic plans and a baseline for monitoring the impact of any revised approach in these areas. A main aim of the Bangladesh survey was to obtain information specifically for the areas defined by the national Control of Iodine Deficiency Disorders project as low performing. Low-performing areas tended to be harder-to-reach areas, border areas, and areas of small-scale seasonal salt production. In Ghana and Senegal, the nationally agreed-upon focus for the surveys was to obtain representative information about areas of small-scale salt production that had been associated with lower household use of iodized salt; therefore, salt-producing areas were selected as a specific stratum.

In Ethiopia, India, Indonesia, the Philippines, and Niger, the surveys were designed to be representative by administrative region; in Tanzania and Uganda, by urban or rural residence. Only the adjusted (weighted) data representative of national, urban, and rural areas are presented for each country in this paper.

The target unit for all surveys was the household. An overview of key survey design features and target sample size, along with the context for each survey, whether it was a specific iodine survey, a health and nutrition survey, or a Fortification Assessment Coverage Toolkit survey, is presented in **Table 1**.

**TABLE 1:** Overview of the survey design for each country^1^

		Target sample size		Sample design		
Country survey context	Year conducted	Total HHs	HHs/PSU	Stratification	Sampling scheme	Wealth or poverty variable
Bangladesh^2^	2015	1512	12	3 strata: urban (including slum), rural low-performing, and rural other	Cross-sectional cluster, based on Multiple Indicator Cluster Survey 2009 sampling frame (PPS)	MPI
					With replacements (99 HHs)	
Ethiopia^3^	2015	4026	11	9 regions and 2 city administrations	Cross-sectional cluster, PPS within strata	Not yet analyzed
					Without replacements	
Ghana^2^	2015	2112	16	4 strata: north, mid, south salt-nonproducing, and south salt-producing	Cross-sectional cluster, PPS within strata	MPI
					Without replacements	
India^2^	2014–2015	6048	12	12 strata: urban or rural by 6 zones: north, northeast, east, west, central, and south	Cross-sectional cluster, PPS within strata	MPI
					Without replacements	
Indonesia^3^	2013	25,000^4^	25	2 strata: urban and rural	Cross-sectional cluster, PPS within strata	Wealth quintile
					Without replacements	
Niger^3^	2014	4320	20	8 administrative regions: Agadez, Diffa, Dosso, Maradi, Mianemy, Tahoua, Tillabéry, and Zinder	Cross-sectional cluster, PPS within strata	Not included
					Without replacements	
Philippines^3^	2013–2014	9813	Varied	17 regions: Ilocos, Cagayan Valley, Central Luzon, Calabarzon, Mimaropa, Bicol Region, Western Visayas, Central Visayas, Eastern Visayas, Zamboanga Peninsula, Northern Mindanao, Davao, SOCCSKSARGEN, NCR, CAR, ARMM, and Caraga	Cross-sectional cluster, PPS within strata	Wealth quintile
					Without replacements	
					Based on replicates of the Philippines Statistics Authority 2003 Master sample	
Senegal^2^	2014	1968	16	3 strata: urban, rural salt-nonproducing, and rural salt-producing	Cross-sectional cluster, PPS within strata	MPI
					Without replacements	
Tanzania^5^	2015	1050	15	2 strata: urban and rural	Cross-sectional cluster, PPS within strata	MPI
					With replacements (9 households)	
Uganda^5^	2015	1101	Mean 16	2 strata: urban and rural	Cross-sectional cluster, PPS within strata	MPI
					Without replacements	

1 ARMM, Autonomous Region in Muslim Mindanao; CAR, Cordillera Administrative Region; HH, household; MPI, Multidimensional Poverty Index; NCR, National Capital Region; PPS, probability proportional to size; PSU, primary sampling unit; SOCCSKSARGEN, South Cotabato, Cotabato, Sultan Kudarat, Sarangani, and General Santos City.

2 Iodine surveys that were fully supported by the Partnership Project (along with the Micronutrient Initiative in Senegal) (11–13; others unpublished).

3 The Partnership Project supported a module for collection and quantitative analysis of iodine in household salt as part of a larger national nutrition survey [Ethiopia, Indonesia, and the Philippines (14)] and a Standardized Monitoring and Assessment of Relief and Transitions survey (Niger).

4 Salt samples from only one-half of all sampled households submitted for analysis.

5 Fortification Assessment Coverage Toolkit surveys that were Global Alliance for Improved Nutrition–supported and implemented with technical support from the US CDC.

Measurement of urinary iodine in ≥1 population group was included in 6 countries, and urinary sodium was also assessed in 2 of those 6. Detailed results for these indicators are not presented here.

#### Overview of survey tools.

All survey instruments contained modules to allow for the classification of residence type (urban compared with rural) and for collection and recording of a household salt sample. The survey tools for 8 countries included modules to assess poverty and SES. However, the specific methodology varied between countries. In Bangladesh, Ghana, India, Senegal, Tanzania, and Uganda, modules were included to calculate the Multidimensional Poverty Index (MPI) score (15, 16), which is being increasingly adopted for use by the UN Development Program (17, 18). A household was classified as being in poverty if the MPI score was ≥0.3 (scale of 0 to 1). Wealth indexes based on the type of composite indicators used in Demographic and Health Surveys (19) were modified to define SES indicators in Indonesia (20) and the Philippines (14). Although each index measures different aspects of poverty and wealth, for the purpose of this paper, the outcome of these 2 methods are both referred to as indicators of SES. The Niger survey (a Standardized Monitoring and Assessment of Relief and Transitions survey that included a short module for salt collection) was designed as a rapid assessment and did not include a composite indicator of wealth or vulnerability to poverty. The Ethiopia micronutrient survey included indicators of wealth; however, these are not yet available as a composite indicator.

#### Survey administration and field procedures.

Interviews were conducted in all selected and consenting households. In all surveys, data were collected by interviewers under the supervision of experienced field supervisors, with coordination and support from technical personnel at the central level. All survey-related personnel were trained before the surveys, and survey tools and procedures were pilot-tested in a typical field setting.

A sample of 20–50 g salt was targeted for collection from all consenting households in each survey, except in Indonesia, in which the aim was to collect 10 g, and in the Philippines, in which ∼100 g of salt was collected. Samples were kept in labeled resealable bags or closed plastic containers and stored in opaque bags or envelopes at room temperature until analysis of iodine content.

In 5 countries (Ethiopia, Ghana, India, the Philippines, and Senegal), data were collected with the use of mobile devices with precoded skips and crosschecks to ensure data quality. In the other countries, data were collected with the use of paper forms. Data quality was ensured by random repeat interviews in most countries; by end-of-day checks and required follow-up by field supervisors; and by validated double data entry with checks for valid ranges, legal values, and consistency (the Niger survey included these validation checks, but did not use double data entry).

#### Indicators of risk and coverage.

Indicators of risk (for access to adequately iodized salt) presented in this paper are confined to an indicator of SES, defined above, and residence type: urban or rural residence was determined by the reference data used to draw the survey sample in each respective country. Rural residence and low SES are considered to be risk factors for low access to adequately iodized salt based on existing evidence of related disparities in household adequately iodized salt coverage (9, 21).

The primary indicator of progress toward achieving optimal iodine nutrition presented in this paper is the coverage of adequately iodized salt, defined as the percentage of households that used salt with ≥15 mg I/kg [the indicator used for global reporting (22)]. The coverage or percentage of households that used noniodized salt (no added iodine) and the percentage of households that used salt with some added iodine, representing all salt that has been iodized, albeit at suboptimal concentrations, are also presented as separate indicators.

To allow for some investigation of the program context for the observed results, information was collected from the Partnership Project national proposals based on country review missions and on other program-related documentation about legislation, salt industry consolidation (an important indicator of the feasibility and sustainability of USI implementation), and key programmatic challenge areas for each country.

#### Determination of salt iodine concentration.

All salt iodine results presented here are based on quantitative analysis of salt iodine while using validated methods. The titration method (23) was used to assess salt iodine content in all surveys except for those in the Philippines, in which the WYD machine was used (Salt Research Institute of China, National Salt Industry Corporation, Tianjin, China, website in Chinese only), and for Tanzania and Uganda, in which the iCheck Iodine device was used (24). The quality of quantitative salt iodine data was ensured in Bangladesh, Ethiopia, Ghana, India, Niger, and Senegal through the implementation of an ongoing internal and external quality assurance system with samples provided from, and results reported to, a third-party laboratory. Salt iodine analysis with the use of the WYD and the i-check was standardized by using measurement of a standard density glass filter (Iodine Standard) to control emitter and receptor settings before each set of measurements, and standard iodized salt samples (1 level for the i-check and 3 levels for WYD) were analyzed at regular intervals to control the measurement process.

The fact that salt iodine data in these surveys were based on quantitative assessment of iodine content means that the relative differences in household coverage with iodized compared with adequately iodized salt can be investigated more reliably than when only semiquantitative methods are used (25).

#### Data analysis.

Data management and analysis for the surveys were conducted by different national and international groups or institutes with the use of different data processing packages; these are presented in **Table 2**. National (and, where required, residence type and SES) data were adjusted for the relative proportion of the population in each stratum. Frequencies and 95% CIs around the frequencies were calculated with the use of these adjusted data. Nonoverlapping 95% CIs were used to determine that a difference between reported coverage frequencies was significant.

**TABLE 2:** Data management and analysis^1^

Country	Institute responsible for data management analysis	Statistical package used
Bangladesh	International Centre for Diarrheal Disease Research, Bangladesh	STATA 13.0 SE; SPSS version 20
Ethiopia	Ethiopian Public Health Institute	SPSS version 16
Ghana	SSC, University of Reading, United Kingdom	SPSS version 22
India	SSC, University of Reading, United Kingdom	SPSS version 22
Indonesia	NIH Research and Development, Ministry of Health	SPSS versions 18, 19, and 20
Niger	National Institute of Statistics, Niger SSC, University of Reading, United Kingdom	SPSS version 22
Philippines	Food and Nutrition Research Institute	STATA 12
Senegal	SSC, University of Reading, United Kingdom	SPSS version 22
Tanzania	CDC	SAS version 9.4
Uganda	CDC	SAS version 9.4

1 SSC, Statistical Services Centre.

Approvals to use the data were obtained from the principal investigator of each survey (USI Coverage Survey Team). Original data were available to the authors through their role as Partnership Project technical support for Bangladesh, Ghana, India, and Senegal, and were provided in summary form by the survey principal investigators or extracted from draft or final survey reports for all other countries. Categories of household salt iodine were determined nationally as noniodized (<5 mg/kg for all countries except for Tanzania and Uganda, in which it was <7.5 mg/kg, based on the analytic limit of detection used, and Ethiopia, in which it was reported as <1 mg/kg), inadequately iodized (for samples considered to be iodized, but at a concentration <15 mg/kg), and adequately iodized (for salt samples containing ≥15 mg/kg). Results are also presented for households that used salt with some added iodine (i.e., salt with iodine above the cutoffs defined for noniodized salt above; other than Ethiopia, this cutoff for some added iodine was >1 mg/kg to try to differentiate between salt with low concentrations of naturally occurring iodine and salt with iodine added during processing).

An analysis was conducted to investigate the relative percentage difference in rural household coverage with both adequately iodized and iodized salt compared with urban coverage, to provide an indication of the degree of relative difference in coverage by residence type. A similar analysis of the relative difference in coverage with adequately iodized and iodized salt for low SES households when compared with coverage in high SES households was done to indicate the degree of relative difference in coverage by SES.

Further details of individual survey design and data management, adjustments, and analysis can be found in the full survey reports (11–13, 20).

Ethical clearance to conduct the surveys was obtained from national or academic institutional review boards in each country. All survey protocols specified informed consent for both the interview and salt sample collection. Individual household identification was made anonymous in all sources of data presented in this paper.

## Results

### Overview of national salt iodization interventions

The basic background information on national salt iodization interventions, presented in **Table 3**, shows that some form of salt iodization is mandatory in all 10 countries and that iodization of food industry salt is included in the legislation in 8 cases, although this is not always recognized, enforced, or monitored in the same way as household salt. Information on the level of salt industry consolidation shows that in 2 countries, ≥80% of nationally available salt is sourced from large- or medium-scale domestic producers (India) or salt iodization processors (the Philippines). In Uganda and Niger, almost all salt is imported. However, in Uganda, the import supply chain is highly consolidated (from Kenya), whereas salt sourced by Niger is from a much more fragmented supply chain (from Ghana, Senegal, Algeria, and other countries).

**TABLE 3:** USI context (legislation, industry consolidation, and challenge areas) for countries in which surveys were conducted^1^

	Legislation for USI			Salt industry consolidation	Identified challenge areas^2^							
Country	Mandatory for household salt	Year (reference)	Includes food-industry salt^3^	Estimated national market from large- or medium-scale producers,^4^%	QA/QC	Legislation regulations	Small-scale producers	Food industry salt	Sustainable potassium iodate supply	Policy/coordination	Awareness (producer, consumer)	Evidence base
Bangladesh	Yes	1989 (26)	No	75	✓	✓	✓		✓	✓	✓	✓
Ethiopia	Yes	2011 (27)	Yes	50	✓	✓	✓		✓	✓	✓	✓
Ghana	Yes	2001 (28)	Yes	40	✓		✓	✓	✓	✓	✓	✓
India	Yes	1997 (29)	Yes	80	✓						✓	✓
Indonesia	Yes	1994 (30)	Yes—decree No—regulations	40–45 (from national production; 50% imported)	✓	✓	✓	✓	✓	✓	✓	
Niger	Yes	1995 (31)	Yes	All imported	✓			✓		✓	✓	✓
Philippines	Yes	1995 (32)	Yes	90	✓		✓	✓		✓	✓	
Senegal	Yes	1994 (33)	Yes	30	✓		✓	✓	✓		✓	
Tanzania	Yes	1994 (34); 2011 (Zanzibar)^5^ (35)	Yes	45 (from national production; 20% imported)	✓		✓	✓	✓	✓	✓	
Uganda	Yes	1997 (36)	Yes	90–95 (imported)							✓	✓

1 QA/QC, quality assurance/quality control; USI, Universal Salt Iodization.

2 Program areas identified for support through national USI review missions at the start of the Global Alliance for Improved Nutrition–UNICEF USI Partnership Project. An additional challenge area for India was to improve access to adequately iodized salt through subsidized distribution systems. Challenge areas for Tanzania and Uganda are based on national USI situational analyses (37, 38).

3 Legislation is for all food-grade (or edible) salt and specifically includes salt for the food industry.

4 Approximate estimates for the percentage of the domestic market share from large- or medium-scale national producers. Large- and medium-scale salt producers are considered to be those with the capacity to produce ≥1000 metric tons salt/y.

5 Legislation for USI was passed in different years for mainland Tanzania (1994) and for Zanzibar (2011).

The programmatic challenges to the achievement of USI identified for support in all 10 countries were in the areas of quality control, regulatory monitoring, and awareness about iodine deficiency and iodized salt along the supply chain from producers to consumers. Other challenge areas included enforcement of regulations, in particular with respect to the food industry and for small-scale salt production, establishing sustainable supplies of potassium iodate, national and subnational coordination mechanisms to effect policies, and a lack of data from which to develop strategies to improve the quality of salt iodization and increase access to all population groups.

### Characteristics of the survey population

The characteristics of the survey population are shown in **Table 4**. Response rates for the household salt samples analyzed varied from 74% in Uganda to 99% in Bangladesh (in which replacement households were used). The national percentage of all interviewed households categorized as vulnerable to poverty (by MPI) varied from 24.5% in India to 57.1% in Senegal. Where wealth indexes were used in Indonesia and the Philippines, 14.5% and 22.3% of households in which salt samples were collected were categorized as being in the lowest wealth quintile, respectively, and 20.3% and 16.9% were categorized as being in the highest wealth quintile, respectively.

**TABLE 4:** Overview of national survey population characteristics^1^

		Households by SES indicator,^3^%			
		MPI score		Wealth quintile^4^	
Country	Response rate, salt samples analyzed,^2^%	Low MPI (nondeprived)	High MPI (deprived)	Lowest wealth index (poorest)	Highest wealth index (richest)
Bangladesh	99.0	56.0	44.0	—	—
Ghana	81.0	52.5	47.5	—	—
India	93.9	75.5	24.5	—	—
Senegal	79.8	42.9	57.1	—	—
Tanzania	77.1^5^	55.0	45.0	—	—
Uganda	74.3	49.0	51.0	—	—
Indonesia	90.3 (45.7)^6^	—	—	14.5	20.3
Philippines	80.9	—	—	22.3	16.9
Ethiopia^7^	80.2	—	—	—	—
Niger^7^	87.4	—	—	—	—

1 MPI, Multidimensional Poverty Index; SES, socioeconomic status.

2 Compared with households targeted for salt collection.

3 Percentage of households with different MPI scores is based on the entire survey sample in all cases except in Indonesia and the Philippines, in which salt was collected from a subset of the national survey sample; therefore, wealth index estimates are only presented for households in which salt was collected.

4 Only data for lowest and highest wealth index are presented.

5 Data for household coverage with adequately iodized salt and any iodized salt from the 2015 Tanzania survey were almost the same with (weighted) inclusion of Zanzibar as for mainland Tanzania alone. Therefore, all Tanzania-related results and discussion in this paper include Zanzibar. The response rate for mainland Tanzania excluding Zanzibar was 81.8%.

6 In Indonesia, 21,741 of the intended 25,000 salt samples were collected for titration. However, only 12,653 samples were submitted for testing, of which results are available for 11,430 (11,430 of 12,653 = 90.3%; 11,430 of 25,000 = 45.7%).

7 No wealth indicator available.

### Household coverage of iodized and adequately iodized salt

**FIGURE 1:**
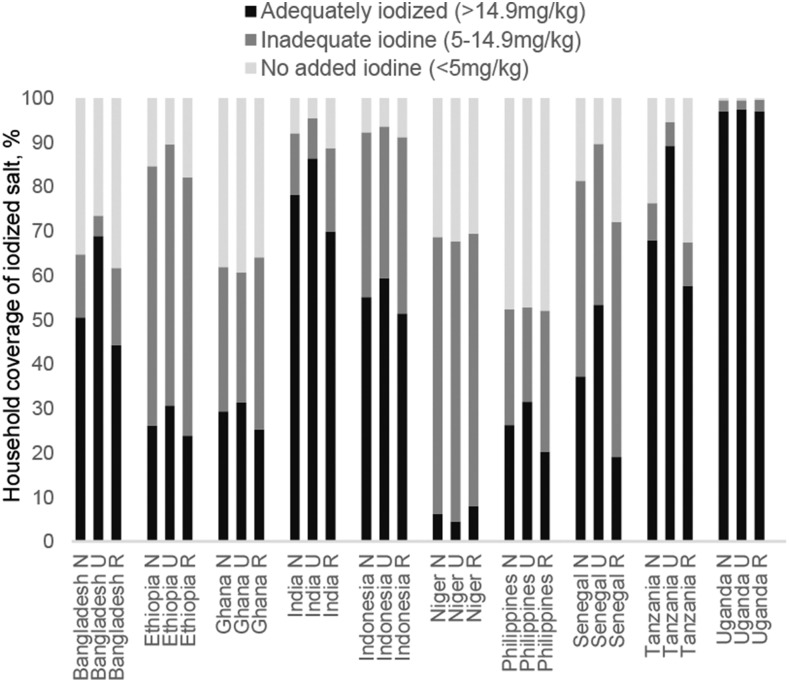
Household coverage with adequately iodized, inadequately iodized, and noniodized salt for N, U, and R areas of 10 countries. National household surveys 2013–2015. For Tanzania and Uganda, the cutoff for some added iodine is ≥7.5 mg I/kg; for Ethiopia, the cutoff is ≥1 mg I/kg. N, national; R, rural; U, urban.

Household coverage with iodized (some added iodine) and adequately iodized salt is shown for each country nationally and by residence type in **Table 5**. The category of salt iodine concentration (noniodized, inadequately iodized, and adequately iodized) is shown also nationally and by residence type, in **Figure 1**. Nationally, household coverage of adequately iodized salt varied from 6.2% in Niger to 97.0% in Uganda. For salt with some added iodine, national household coverage varied from 52.4% in the Philippines to 99.5% in Uganda.

**TABLE 5:** Overview survey results for percentage household coverage with iodized and adequately iodized salt

		Household coverage for iodized salt,^1^%	
Country and stratum or zone	Total salt sample (unweighted), *n*	Some added iodine^2^	Adequately iodized (≥15 mg/kg)
Bangladesh			
National	1498	64.7 (58.8, 70.1)	50.5 (42.1, 58.9)
Urban	501	73.4 (59.3, 83.9)	68.9 (55.8, 79.5)^a^
Rural	997	61.7 (55.1, 68.0)	44.3 (34.5, 54.6)^b^
Ethiopia			
National	3229	84.6 (not available)	26.1 (not available)
Urban	1077	89.5 (not available)	30.6 (not available)
Rural	2152	82.1 (not available)	23.8 (not available)
Ghana			
National	1569	61.9 (57.3, 66.2)	29.3 (25.3, 33.6)
Urban	997	60.7 (54.6, 66.6)	31.4 (26.5, 36.9)
Rural	572	64.0 (57.5, 70.0)	25.2 (17.7, 34.5)
India			
National	5682	92.0 (90.7, 93.1)	78.1 (76.2, 79.9)
Urban	2838	95.4 (94.0, 96.4)^a^	86.4 (84.4, 88.1)^a^
Rural	2844	88.6 (86.2, 90.5)^b^	69.8 (66.6, 72.9)^b^
Indonesia			
National	11,430	92.3 (91.9, 94.1)	55.1 (54.4, 55.9)
Urban	6172	93.6 (93.1, 94.1)^a^	59.3 (58.2, 60.4)^a^
Rural	5258	91.2 (90.6, 91.8)^b^	51.4 (50.3, 52.4)^b^
Niger			
National	3772	68.6 (not available)	6.2 (not available)
Urban	2107	67.7 (not available)	4.5 (not available)
Rural	1665	69.4 (not available)	8.0 (not available)
Philippines			
National	7984	52.4 (50.3, 54.5)	26.2 (24.4, 28.0)
Urban	3492	52.8 (49.6, 56.0)	31.5 (28.6, 34.4)^a^
Rural	4492	52.0 (49.1, 54.9)	20.2 (18.1, 22.3)^b^
Senegal			
National	1566	81.3 (77.3, 84.8)	37.2 (32.2, 42.4)
Urban	474	89.6 (84.3, 93.2)^a^	53.3 (46.0, 60.4)^a^
Rural	1092	72.0 (65.3, 77.9)^b^	19.0 (12.8, 27.2)^b^
Tanzania			
National	810	76.3 (68.6, 84.0)	67.9 (58.5, 77.4)
Urban	331	94.5 (89.8, 99.1)^a^	89.2 (83.7, 94.6)^a^
Rural	479	67.4 (56.3, 78.6)^b^	57.6 (44.0, 71.3)^b^
Uganda			
National	818	99.5 (99.0, 100.0)	97.0 (94.3, 99.8)
Urban	389	99.5 (98.8, 100.0)	97.4 (95.9, 99.0)
Rural	429	99.6 (98.9, 100.0)	97.0 (95.3, 98.6)

1 Values are % (95% CI). CIs were not available for all data sets; therefore, these are not shown for Ethiopia and Niger. Labeled values in a row without a common superscript letter are significantly different in coverage by residence type for that country, based on nonoverlapping 95% CIs.

2 “Some added iodine” was defined as salt with ≥5 mg I/kg for all countries except Tanzania and Uganda, in which it was defined as salt with ≥7.5 mg I/kg, and Ethiopia, in which it was defined as salt with ≥1 mg I/kg.

#### Adequately iodized salt by residence type.

Household coverage of adequately iodized salt was generally lower in areas of rural residence than in urban areas (except for in Niger, in which coverage in urban households was slightly lower). The 95% CIs around the percentage household coverage estimate (available for all countries except Ethiopia and Niger) shown in Table 5 indicate that household coverage of adequately iodized salt was significantly higher in urban than in rural areas in Bangladesh, India, Indonesia, the Philippines, Senegal, and Tanzania. Although 95% CIs were not available around the coverage estimates from Ethiopia, household coverage of adequately iodized salt was 22% lower in rural households than it was in urban households. The largest relative difference was observed in Senegal, in which coverage of adequately iodized salt was 64% lower in rural households than it was in urban households.

#### Salt with some added iodine by residence type.

Household coverage of salt with some added iodine was significantly higher in urban than in rural areas for India, Indonesia, Senegal, and Tanzania (Table 5). The largest relative difference was observed in Tanzania, in which household coverage of salt with some added iodine was 29% lower in rural households than it was in urban households. In Senegal, the relative difference for rural coverage compared with urban coverage was 19.6%. In Bangladesh, the relative difference was 15.9%; however, the wide 95% CI around the point estimate for household coverage of salt with some added iodine in urban areas resulted in a high degree of overlap with the 95% CI around the rural point estimate meaning that the difference was not significant. For Ethiopia, coverage of salt with any added iodine was 8% lower in rural households than was coverage in urban households, similar to the relative difference found for India (7.1%).

#### Adequately iodized salt by SES.

**FIGURE 2:**
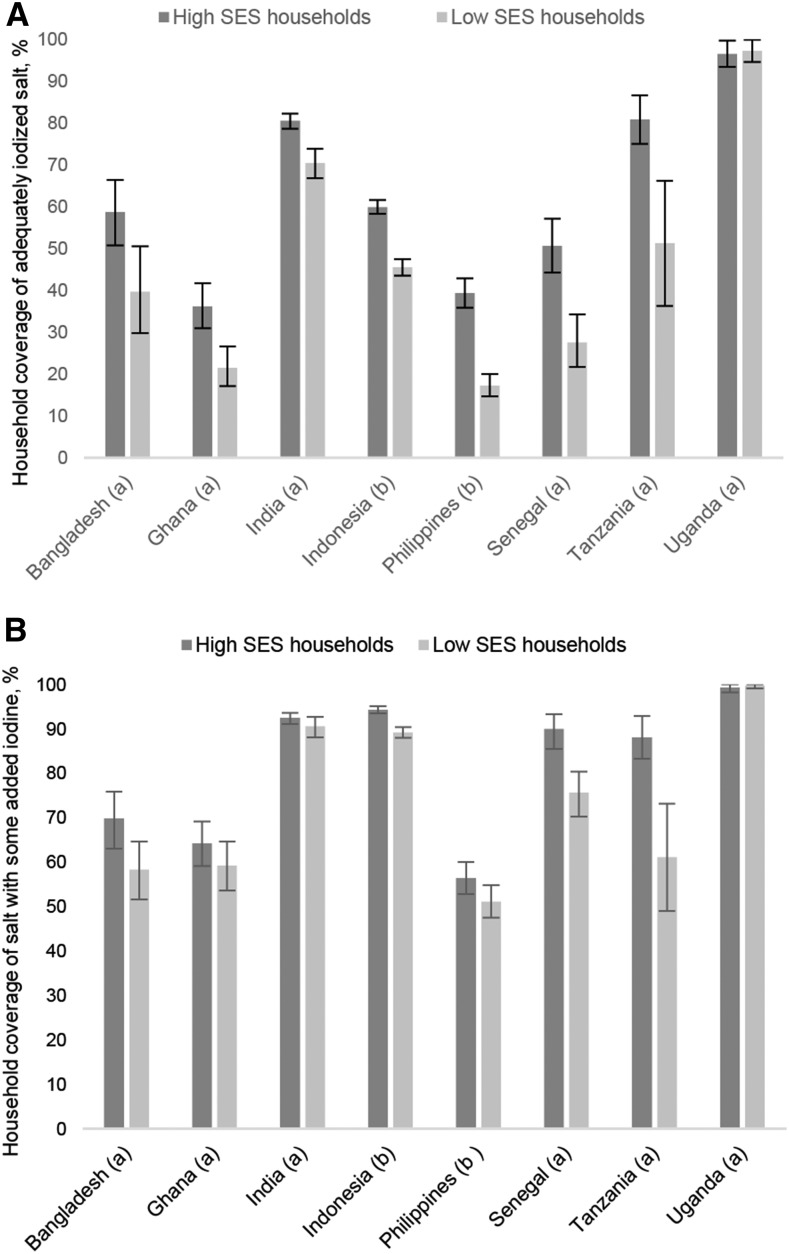
Household coverage with adequately iodized salt (≥15 mg/kg) (A) and with iodized salt (some added iodine) (B) by SES; national household surveys 2013–2015. Values are percentages with 95% CIs around the estimates. SES was assessed in different countries by Multidimensional Poverty Index (a) or highest and lowest wealth quintile (b). For Tanzania and Uganda, the cutoff for some added iodine (B) is ≥7.5 mg I/kg; for all other countries shown here, the cutoff is ≥5 mg I/kg. SES, socioeconomic status.

Except for Uganda, in all countries in which SES data were available (this excludes Niger and Ethiopia), the percentage of households that were using adequately iodized salt was significantly higher (nonoverlapping 95% CIs) in households with a higher SES (low MPI or highest wealth quintile) than in this with a lower SES, as shown in **Figure 2A**. This association was highest in Senegal and the Philippines, in which the relative coverage with adequately iodized salt in low-SES households was 46% and 56% lower, respectively, than coverage in high SES households. In Bangladesh, Ghana, and Tanzania, low-SES households had 25–40% lower coverage of adequately iodized salt relative to coverage in high-SES households. In India and Indonesia, household coverage of adequately iodized salt was 10–25% lower in low-SES households than it was in high-SES households.

#### Salt with some added iodine by SES.

Results for salt with some added iodine revealed smaller differences in household coverage by SES, as shown in Figure 2B. The difference in household coverage of salt with some added iodine by low and high SES was only significant (nonoverlapping 95% CIs) in Indonesia, Senegal, and Tanzania. Tanzania was the only country in which the relative difference (coverage in lower-SES households relative to coverage in high-SES households) was approximately the same (30.6%) as was observed for adequately iodized salt (36.6%).

## Discussion

This review shows that coverage with adequately and any iodized salt varies considerably between and within countries. Results illustrate considerable national progress and achievement in Uganda and India. Substantial progress has also been made in Ethiopia, in which data from 2011 found <16% household coverage of salt with some added iodine (39). However, results also highlight persistent inequities in household access to adequately iodized salt, identified and highlighted more generally in a UNICEF review of 2006–2011 survey data (9).

Of the 10 countries included in this paper, only Uganda had achieved USI (≥90% household coverage with adequately iodized salt), and this was observed both nationally and subnationally, in urban and rural areas. It will be important for the government of Uganda to monitor and sustain this successful achievement and to share lessons with other countries. In 5 of the 10 countries, more than one-half of the population remains at risk of iodine deficiency because of limited access to adequately iodized salt (<50% national household coverage in Ethiopia, Ghana, Niger, the Philippines, and Senegal).

In general, national and subnational areas found to have higher coverage of adequately iodized salt were associated with a higher level of industrial consolidation and mechanization of the salt supply (i.e., India, Uganda, and urban areas of Bangladesh and Tanzania). In line with this, national survey reports for Bangladesh (11), Senegal (13), and Ghana (personal communication from EF Amoaful, Ghana Health Services, 2016) indicate that subnational strata representing areas of extensive small-scale salt production had particularly low coverage of adequately iodized salt (<26% of households). The low coverage is suggested to be the result of lower technical capacity of small-scale salt producers to iodize salt, along with the increased challenges to establish effective quality assurance and regulatory monitoring of iodization in areas of widespread artisanal salt production (J Gorstein, unpublished results, 2015) (40, 41).

### Inequity in access to adequately iodized salt.

The observations in this paper with regard to lower access to adequately iodized salt in rural and lower-SES (high MPI, lower wealth quintile) populations is in agreement with previous reports (9, 21). Access to adequately iodized salt at the household level is generally dependent on 2 factors: product availability and affordability. Differences in access to adequately iodized salt reported here by residence type and SES could be the result of one or both of these factors. Further investigation, including analysis of retail availability and pricing of quality-assured iodized salt, and of consumer purchasing practices (e.g., preference for packaged instead of loose salt or vice versa), would be required to understand which factors have greater influence in different contexts. It is important to note that the aim of USI is to ensure quality-assured iodization of all salt for human and animal consumption, regardless of grain type and packaging. In a situation in which USI is fully implemented, as demonstrated in Uganda, access to adequately iodized salt would become equitable, regardless of consumer preferences and affordability.

Results from this work provide important insights to guide future national strategies to achieve USI. For example, in Ghana and the Philippines, the relative difference in household access was much more pronounced by SES than by residence type, suggesting that adequately iodized salt, inadequately iodized salt, and that with no added iodine may all have been readily available in both urban and rural areas. In such a situation, it could be hypothesized that although adequately iodized salt is available, lower-SES households generally have greater access to lower-priced, lower iodization–quality salt. In some cases, this may potentially be salt sourced at the point of production, before any iodization step that may take place, as indicated in the survey reports for Bangladesh, Ghana, and Senegal. In Bangladesh, Senegal, and Tanzania, the level of notable disparity in coverage by residence type that is similar to that by SES could suggest that availability of adequately iodized salt (generally packaged and more expensive in these countries) is linked to urban residence type.

Inequities in coverage (by residence type and by SES) almost disappeared in most countries when investigated by access to salt with some added iodine. This fact suggests that the quality-assured, adequately iodized salt product was well defined and recognizable within national markets (e.g., fine grain or packaged) and most likely of higher price, whereas inadequately iodized salt and noniodized salt were possibly more similar to each other in perceived characteristics and price and could have been bought interchangeably without the consumer necessarily being aware of any difference. Tanzania was the only country in which the level of disparity in access (by residence and SES) remained in the same range for salt with some added iodine compared with adequately iodized salt. This finding may be a reflection of the fact that a very low percentage of households (8.4%) in Tanzania were accessing inadequately iodized salt.

Many of these hypotheses are supported in principle by results and recommendations presented in the respective national survey reports. Although beyond the scope of this paper, it will be important to conduct a more detailed investigation of factors associated with salt iodine content and consumer access in order to better understand the barriers to achievement of USI and to further develop evidence-based strategies.

These findings confirm that the previously identified challenge areas related to strengthening regulatory monitoring and enforcement of legislation to achieve high-quality salt iodization with appropriate packaging have not been fully ameliorated. This strategy remains as a recommended focus to accelerate progress toward achieving and sustaining optimal iodine status through USI, both nationally and subnationally, in Bangladesh, Ethiopia, Ghana, Indonesia, Niger, the Philippines, Senegal, and Tanzania. These challenge areas remain of particular importance in relation to small-scale salt-producing areas, in which industry modernization and consolidation, along with strengthened quality control and regulatory monitoring, will be crucial factors for successful improvements in iodization quality (42, 43).

The relatively high household coverage and tight 95% CIs around the point estimates for India indicate that strong quality assurance procedures are being implemented. The national survey report recommendations focus on reaching the last 20% of households through further consolidation of the salt industry, in parallel with specific strategies to reach marginal populations, e.g., by ensuring the supply of quality-assured iodized salt through the public distribution system.

Niger had by far the lowest household coverage of adequately iodized salt of the 10 countries included here, suggesting that >90% of the population could be at risk of iodine deficiency through lack of access to well-iodized salt. The country relies almost entirely on imports of edible salt, mainly from Ghana and Senegal; therefore, progress in Niger will be, to some extent, dependent on stronger monitoring and enforcement of regulations within those 2 countries. However, there is a parallel and urgent requirement to strengthen the capacity of national regulatory monitoring personnel to enforce existing legislation at the point of salt import. An additional factor in Niger is that salt from Algeria, imported as noniodized salt for industrial purposes, has leaked into the domestic market for human consumption.

It should be kept in mind that percentage household coverage figures for adequately iodized salt do not necessarily represent the impact of the problem in terms of population numbers. This can be demonstrated by comparing estimates for household coverage for adequately iodized salt and the population yet unreached in Niger (in which <10% coverage reflects ∼18.6 million people without access to adequately iodized salt) with India (in which there has been remarkable progress and almost 80% coverage, yet the remaining 20% represents ∼287 million people).

### Strengths and limitations of the survey methodologies and review.

The main strengths of the studies presented here are the following: *1*) the quantitative analysis of salt iodine content (with external quality assurance of the results in most instances), providing reliable estimates of the quality of iodized salt that will help inform the strategy for USI and future coverage surveys; *2*) stratification to provide programmatically relevant representative data in some countries (data by strata not shown); and *3*) the incorporation of poverty or wealth indicators, reported for 8 of the studies here, that allowed for subgroup analysis of household coverage by the known risk category of low SES.

The difference in survey design between countries limited the level of between-country comparison and within-country disaggregation that could be conducted.

Household coverage of iodized and adequately iodized salt may provide only limited information about total dietary salt and iodized salt intake, and other major sources of dietary salt are not represented in this review, although some related food frequency data were collected as part of surveys in Ghana, India, Indonesia, and Senegal.

### Implications for future national strategies and for monitoring and assessment of USI.

In countries in which inequities in access to adequately iodized household salt have been identified, an innovative review is recommended to develop and implement targeted strategies to ensure access for communities in harder-to-reach areas, small-scale salt producing areas, areas with higher vulnerability to poverty, and other groups with lower access to quality-assured iodized salt. These recommendations echo those from a recent analysis of changes in household salt iodine over time in relation to indicators of equity (21).

In an environment of increasing consumption of processed foods, condiments, and foods prepared outside the home, there are many other dietary sources of (potentially iodized) salt, particularly in urban areas, in which diets tend to be more diversified (44–47). As a result, there are increasing international calls for a re-evaluation of the use of household coverage with adequately iodized salt as the sole indicator to measure national progress toward achieving and sustaining optimal iodine status through USI (48). Other sources of dietary salt, potentially iodized, should be considered when making any assumptions about the adequacy of population iodine intake from USI. For example, in the Ghana and Senegal surveys, the outcome from additional food consumption modules, interpreted in conjunction with research on the retention of iodine in bouillon (49), suggest that if all bouillon was produced with the use of adequately iodized salt, it would contribute significantly to iodine intake across population groups in both countries, including in areas in which coverage of adequately iodized household salt was found to be low.

The degree to which nonhousehold salt contributes to total dietary salt intake would be expected to vary between and within countries according to national food industry structure and distribution channels, population access to markets, and differences in dietary practices. It is understood that it is increasingly important to improve national knowledge of these factors. It is suggested here that strengthening the monitoring and quality assurance systems for iodized edible salt should include major food and condiment processing industries. In addition, future survey tools should include a measure of assessment for commonly consumed centrally processed foods and condiments known to contribute to salt intake.

## Conclusion

Good progress has been made toward high levels of household access to adequately iodized salt throughout India and Uganda and in urban areas of Bangladesh and Tanzania. Substantial increases in household coverage of adequately iodized and iodized salt have also been achieved in Ethiopia compared with coverage from the past 5 y. At the subnational level in Bangladesh and Tanzania and at all levels in Ghana, Indonesia, Niger, the Philippines, and Senegal, progress has reached a plateau, and innovative strategic review is recommended. Salt industry modernization, consolidation, and related improved iodization and quality control capacities, along with strengthened regulatory monitoring, have proven to be effective strategies to improve equity of access to quality-assured iodized household salt in some countries. Any revised strategy should also incorporate plans to ensure legislation and enforcement of the use of quality-assured iodized salt in the food industry so that all population groups can access quality-assured iodized salt regardless of dietary practices.

The survey and data analysis methods discussed in this paper highlight the importance of designing assessments to identify population groups with poor or no access to adequately iodized household salt, along with potential causal factors. Analysis and interpretation of the program to achieve and sustain optimal iodine nutrition in relation to these components, together with information on the use of quality-assured iodized salt in the food industry, will result in a better understanding of program achievements and remaining challenges.

## Footnotes

9 Abbreviations used: MPI, Multidimensional Poverty Index; SES, socioeconomic status; USI, Universal Salt Iodization.
